# “Refined Balloon Pulmonary Angioplasty” May Need to Be Refined

**DOI:** 10.1016/j.jacadv.2023.100550

**Published:** 2023-08-09

**Authors:** Benjamin L. Magod, Charlie Quinn, S. Christopher Malaisrie, Michael J. Cuttica, Daniel Schimmel

We would like to congratulate Bashir et al^1^ on their excellent work describing their experience with refined balloon pulmonary angioplasty (BPA) in chronic thromboembolic pulmonary hypertension. Utilizing pressure wire guidance and limiting distal mean pulmonary artery pressures to under 35 mmHg, low complication rates were reported with hemoptysis in 4.7% of BPA sessions and 2.6% of patients requiring intubation.

Prior studies reported higher complication rates, with hemoptysis in 5.6 to 14% of BPA sessions and intubation in 2.5 to 5.5% of patients.^2^^,^^3^ Other studies, which did not routinely use pressure wire guidance except during initial sessions, note a significant decrease as much as in half in complications rates over time.^2^^,^^3^ While Bashir et al^1^ demonstrated fewer complications, the question remains: is the “refined BPA” technique integral to achieving lower complication rates?

At our center, pressure wire guidance is uncommon. We identify pressure gradients via a monorail, sensor-tipped micro pressure catheter to interrogate vessels if a question remains about the residual physiologic severity of a lesion. This method allows the operator to use typical workhorse wires which are more easily maneuvered and potentially more durable. The routine use of a pressure wire may increase procedure time, limit the number of vessels per session, and increase the number of sessions needed to achieve significant improvement. Through our institution’s initial 42 patients and 152 BPA sessions, hemoptysis occurred in 6.5%, and no patients required intubation. Based on this experience, it is these authors' opinion that the refined technique and routine use of pressure wire guidance may not be necessary to achieve lower complication rates.

As Bashir et al^1^ acknowledge, their cohort had less improvement in invasive hemodynamic parameters. While this may be due to incomplete BPA treatment course, patient population factors, or operator experience, it points toward an assumed balance point between hemodynamic response and risk of complications. Further study of BPA techniques will help to elucidate which factors may improve the safety and efficiency of this growing treatment option for chronic thromboembolic pulmonary hypertension.
